# Understanding tumor heterogeneity as functional compartments - superorganisms revisited

**DOI:** 10.1186/1479-5876-9-79

**Published:** 2011-05-27

**Authors:** Thomas GP Grunewald, Saskia M Herbst, Jürgen Heinze, Stefan Burdach

**Affiliations:** 1 Department of Pediatrics, Klinikum rechts der Isar, Technische Universität München, Kölner Platz 1, 80804 Munich, Germany; 2 Laboratory of Functional Genomics and Transplantation Biology, Children's Cancer Research and Roman Herzog Comprehensive Cancer Center, Klinikum rechts der Isar, Technische Universität München, Kölner Platz 1, 80804 Munich, Germany; 3 Medical Life Science and Technology Center, TUM Graduate School, Technische Universität München, Boltzmannstrasse 17, 85748 Garching, Germany; 4 Institute of Human Genetics, University of Regensburg, Franz-Josef-Strauss-Allee 11, 93053 Regensburg, Germany; 5 Biologie I, University of Regensburg, Universitätsstraße 31, 93040 Regensburg, Germany

## Abstract

Compelling evidence broadens our understanding of tumors as highly heterogeneous populations derived from one common progenitor. In this review we portray various stages of tumorigenesis, tumor progression, self-seeding and metastasis in analogy to the superorganisms of insect societies to exemplify the highly complex architecture of a neoplasm as a system of functional "castes."

Accordingly, we propose a model in which clonal expansion and cumulative acquisition of genetic alterations produce tumor compartments each equipped with distinct traits and thus distinct functions that cooperate to establish clinically apparent tumors. This functional compartment model also suggests mechanisms for the self-construction of tumor stem cell niches. Thus, thinking of a tumor as a superorganism will provide systemic insight into its functional compartmentalization and may even have clinical implications.

## Introduction

Cooperation and division of labor are thought to explain many of the major transitions in evolution, in which several simple units form a more complex group [1,2]. When conflict among their constituents is resolved or sufficiently suppressed, such higher biological entities achieve "organismality" at a higher level, i.e., they interact with other such entities as "individuals" [3]. Major transitions are the evolution from independently replicating oligonucleotides into genomes, from prokaryotes to eukaryotes, and from unicellular to multicellular organisms. Another major transition, in which emergent properties arising from cooperation and division of labor are particularly obvious, is the origin of the social insects from solitary organisms. The nests of social insects - ants, termites, and honeybees - consist of hundreds or thousands of individuals, which appear to interact so smoothly and complementarily that the society as whole has been referred to as a "superorganism," in analogy to the well-functioning organism of a multicellular animal [4-10].

Superorganisms are societies composed of specialized reproductives (queens and, in termites, kings) and non-reproductive castes. Workers are fully dedicated to support the royal reproductive caste in an altruistic fashion - that is, they normally follow epigenetically programmed algorithms to fulfill their self-sacrificing behavior of brood care, foraging, and colony defense and in this way increase the reproductive success of the queens (and kings). Rather than directly transmitting copies of their own genes via their own offspring, workers indirectly maximize their fitness via the offspring of the reproductives, to whom they are usually closely related [4,11-14].

Many superorganisms change their environment radically by constructing nests with microclimate control or by connecting them with durable food sources by carefully maintained trails. Some species enrich their food by growing fungi or herding sugar-producing insect symbionts, and others pillage "slaves" from neighboring ant nests during well-organized raids [5,6,15]. This all requires closely controlled cooperation among individuals behaviorally or morphologically specialized for different tasks. Though the gene is the ultimate unit of selection, the insect society as a whole has become target of selection and may be envisaged as the "extended phenotype" of the reproductives' genes [16]. Selection may therefore optimize caste demography, patterns of division of labor, and communication systems at the colony level.

A nascent colony has to overcome several barriers to thrive and expand: young queens or fragments of mature societies must locate an adequate nesting site, the workers have to find and collect nutrients, establish home territories, defend the nest against enemies, and care for the helpless young. The society as a whole may respond flexibly to inductive stimuli either because individuals switch tasks in an opportunistic fashion or because more individuals specialized for a particular task are produced [17-20]. Division of labor in a superorganism ultimately relies, at least in part, on self-organization with positive and negative feedback cycles and usually lacks control by a still higher-level system [17,19,21].

In analogy, there has been great progress in the understanding of solid neoplasms as highly heterogeneous organ-like tissues with a hierarchical cellular organization [22]. Although all cells within a tumor are most likely derived from one common ancestor [23], they differ substantially in shape and function rather than being clonal monocultures [24,25]. Recent data suggest that a solid tumor contains quiescent cells [26] that maintain a stable functioning tumor despite external perturbations by therapy [27]. Those cells are likely not mere hibernating bystanders but rather differentiated cells that actively promote proliferation of their clonemates in accomplishing growth-fostering functions. These may include angiogenesis, immunoediting and construction of an advantageous microenvironment to shelter the tumor stem cells (TSCs) [28-30]. The functional variety of these diversely differentiated tumor cells resembles phenomena seen in superorganisms of social insects.

As indicated above, cooperation among biological entities and subsequent specialization of individuals for specific tasks (division of labor) are general, wide-ranging, and efficient phenomena in evolution [1]. In analogy to these major transitions, and in particular in analogy to the superorganism, the principle of division of labor may also apply in the hierarchical self-construction of neoplasias as complex organ-like tissues. In the following chapters we will propose a model for the self-construction of TSC niches and explain how the thinking of solid tumors as superorganisms may have relevance to the development of novel therapeutic approaches against cancer.

## Clonal and functional relationships of solid neoplasms and superorganisms

It is a widespread consensus that most human tumors are monoclonal growths descending from single progenitor cells [31,32] that - through several rounds of mutations and selection - overcome the constraints imposed by intercellular competition [33,34]. Although this linear cancer progression model is supported by sound and recent evidence, it is still unclear how a nascent tumor might manage to prepare the ground for ongoing growth and how an already established tumor might benefit from tumor heterogeneity (for review see [35] and references therein), which is often observed in specimens of large tumors [36]? Moreover, as most tumors are quite advanced when detected comprising a billion or more cells [32], late stages of tumor development are far better understood than initial events. Yet, these initial events are likely to be crucial for tumor progression [24,37].

We therefore wonder what mechanisms govern the self-assembly of a nascent tumor and what factors shape its continuous development into a heterogeneous organ-like structure?

We approach these questions from a sociobiological perspective and model how principles of division of labor as seen in social insects might operate within a solid tumor to accomplish the needs of ongoing tumor growth:

In our functional compartment model, solid neoplasms are hierarchically organized and like superorganisms consist of different compartments or "castes" that are epigenetically (and in the case of cancers possibly also genetically) specialized for certain tasks. One compartment specializes in reproduction (TSCs), others in foraging (angiogenesis) and still others contribute to the tumor's logistics and expansion (tissue invasion, vascular access). Although only one compartment is *de facto *reproductive, the cooperative (inter-)action of all compartments is essential for the fitness of the solid tumor as a whole.

In social insects, the queen's ovaries harbor the colony-forming "stem cells" that produce rapidly proliferating oocytes - the stem cell's closest progeny - which will develop into offspring that support further upgrowth of the colony. Workers, the "somatic units" of a superorganism, perceive and interact with the environment to ensure nutrient supply and to shelter the queen and thus the stem cells. Defensive castes destroy the environment's "immune system" and protect the colony from external attacks. Specific workers are first to invade and explore uncertain terrain and recruit specialized workers (foragers) that modify the microenvironment to access the colony supporting nutrients [5,6].

In analogy, the TSC concept, which was first indicated in the late 19^th ^century [38], states that only a few scattered cells within a neoplasm can give rise to progeny [25] through infrequent asymmetric cell division [39].

Although TSC have not yet been identified in some tumor entities, there is compelling evidence that many cancers including breast, colon and brain cancer follow a hierarchical TSC model [25,40-42]. In these tumors the rapidly proliferating progeny of the TSCs gradually casts off stem cell traits like self-renewal and multi-potency while simultaneously acquiring defined functional properties through (incomplete) differentiation [25]. This most likely happens due to activation and maintenance of distinct gene expression signatures upon stimuli received from other tumor cells and/or the microenvironment [25,43]. In the TSC's vicinity part of this non-tumorigenic progeny forms a shelter often referred to as the "TSC niche" [44]. This niche resembles a "breeding chamber" stocked with cells, which have specialized in providing factors that prevent differentiation and thus maintain the stemness of the TSC and ultimately the colony's survival [44]. Like in supercolonies of ants, such as the odorous house ant *Tapinoma sessile *[45] that split and reunite again also solid tumors are enriched by recirculating TSCs through cancer self-seeding [46]. An overview of the functional relationships of solid neoplasms and superorganisms is given in Table 1.

**Table 1:** functional relationships of superorganisms and solid neoplasms

Feature	Superorganism	Solid neoplasm
Sociobiological aspect	Sociogenesis: growth and development of the colony	Tumorigenesis: growth and development of the tumor
Reproduction and self-renewal	Queen (foundress)	Tumor stem cells (TSCs)
Specialization for housekeeping work	Worker caste (non-reproductive)	Non-TSC (progeny = limited proliferation, no tumor-initiation ability)
Protection from intruders	Specialized defensive castes: alarm-defense communication, colony recognition labels, camouflage and pheromone repellants	Secretion of anergy inducing cytokines Downregulation of major histocompatibility complexes (MHC)
Communication and interaction among colony members	Pheromones, visual, auditory and haptic signals	Paracrine hormone and cytokine communication, direct cell-cell contact
Shelter and microclimate control	Nest construction	Induction of fibrosis High intratumoral hydrostatic pressure
Habitat	Ecosystem	Organism
Cargo flux and circulatory system	"Ant highways"	(Neo)-angiogenesis Angiogenic mimicry
Driving force for adaptation	Natural selection	Intercellular competition and selection, immunoediting and genetic instability
Multi-colony-formation (inter-group-competition)	Supercolonies Budding and fusion of individual colonies with the supercolony	Symmetric cell division and formation of new TSCs Cancer self-seeding
Colony founding	Queen flight	Metastasis of TSCs

## Evolution of division of labor

Queens that could produce progeny with traits of parental care uncoupled from reproduction [47-49] managed to propagate their genes better than those who could not. A similar principle might also apply to other biological entities exposed to similar selection pressures. In social insects, the evolution of highly cooperative societies from solitary insects presumably needed millions of years due to relatively long generation time of individuals and the relatively low rate of genetic variation. In contrast, due to inactivation of pro-apoptotic factors and DNA-repair mechanisms, most cancer cells suffer from great genomic instability, which dramatically accelerates the evolution of neoplasias [32,34,37,50,51]. However, most cancer cells (>99.8%) are believed to acquire disadvantageous features and to go extinct before establishing a tumor [24,32,52].

Given that tumorigenesis requires acquisition of multiple mutations during a period of many years, stem cells are - due to their long life span - reasonable candidates for the accumulation of mutations ultimately resulting in malignant transformation [32,53]. In addition to their long life span, stem cells are able to generate full lineages of differentiated cells, thereby perpetuating mutations through uncontrolled clonal expansion [32,37]. Multiple studies suggest that neoplasias originate from stem cells or cells that have gained stem cell properties [25,32,54-56]. These tumorigenic cells, the TSCs, are believed to be the driving force in tumor progression and a possible cause of tumor heterogeneity [25,52]. During tumorigenesis some TSCs will gain positive features by mutation, survive, and propagate this survival benefit to their progeny [24,25,37]. Yet, it s still unclear why a TSC gives rise to differentiated daughter cells, which have lost the ability of unlimited self-renewal, and what kind of selective advantage this process could have for the overall fitness of the tumor (for review see [54] and references therein)?

Our model of solid tumors as superorganisms would predict that in the very early phase of a solid tumor all TSCs, albeit rare, would compete with their own progeny for limited space and resources [24,34,52,57,58] unless they manage to propagate traits of "parental care" and cooperation to their offspring. Hence, initially a TSC may need to compete with both: other TSCs and their progeny and with its own offspring [24,33,34,52,57-59]. Its great genetic instability is even likely to aggravate the TSC's struggle within intercellular competition, because it may lead to acquisition of negative traits that undermine cooperation and thus are deleterious for the TSC itself. Put in another way, a nascent tumor is exposed to several selective pressures arising from inter-cellular competition for limited space and nutrients and from the host's immune system as discussed later.

However, some TSCs may by chance manage to propagate epigenetically and/or genetically fixed traits [60] of parental care and cooperation to its non-TSC offspring during asymmetric cell division. In this scenario, the scale of intercellular cooperation would be larger than the scale of intercellular competition. Accordingly, TSCs that produce non-TSCs with a high degree of cooperation should disperse and outcompete those lacking a similar degree of cooperation, because the non-TSCs carrying traits of parental care and cooperation would now alter the environment in a way that makes TSC survival and proliferation more likely, that is, they set-up a well-organized novel tissue with its own internal homeostasis - a so-called TSC niche.

This niche would enhance the overall fitness of the TSC and its progeny for inter-group competition between different TSCs and their progeny. This implies that there might exist mechanisms by which clonemates of one TSC might recognize each other while cooperating. Hence, tumor progression in its microenvironment is, what we believe, similar to the evolution of a superorganism through natural selection in its ecosystem.

**Figure 1:**
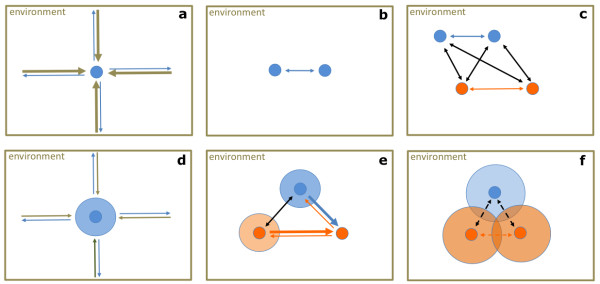
**Selection pressure and evolution of social organisms**: **A) **Each individual cell or organism is embedded in an environment, and both impose constant selection pressure in terms of harmful effects on each other (arrows; the width of the arrows corresponds to the strength of the executed selection pressure). **B) **Non-social individuals of the same generation compete with each other for resources. For reasons of clarity the selection pressure of the environment is not depicted albeit constantly present. **C) **Non-social individuals of proximate generations (parental, blue; F1, orange) also compete all with each other (inter-individual selection). **D) **Social individuals can reduce the inter-individual selection pressure by propagating "altruistic genes", and hence can cope better with the environment (see arrows; the circle resembles the colony). **E) **Colonies of social individuals may drive non-social individuals to go extinct, although they compete with each other (inter-group selection). In social insects, genes engendering cooperation, and specifically the developmental plasticity needed for an efficient division of labor, will be selected because cooperative groups can either outcompete less cooperative groups and/or cooperation allows persistence in otherwise inhospitable environments. **F) **In addition, individuals and their colonies may cooperate to reduce inter-colony selection pressure, as seen in supercolonies of social insects and in solid tumors composed of thousands of tumor stem cells (TSCs) and their inter-cooperating progeny (depicted as overlapping circles).

Most advanced neoplasms are likely to consist of multiple TSCs and their corresponding non-TSC offspring [32,37]. These multiple TSCs are thought to be derived form one common ancestral TSC (referred to as the "one renegade cell") [23], but may, after some time of tumor progression, differ from each other due to epigenetic and/or genetic mutations acquired by each TSC individually [32,37]. In analogy, a small number of so-called "unicolonial" social insects more or less completely lack colony borders. This greatly reduces inter-colony competition and increases the ecological success of such invasive species. It is debated whether unicoloniality is a consequence of the unhindered growth of founding colony after a single introduction event associated with the depletion of diversity in genetic odor cues during the invasion of new habitats [61] or an adaptive response to the new environment [62] (Figure 1).

Hence, according to our functional compartment model a TSC needs to propagate traits of division of labor that are exclusively activated in its progeny, because a TSC cannot functionally differentiate and maintain at the same time its stem cell character that is by definition an undifferentiated state. *Vice versa*, the functional differentiation of the TSC's progeny is acquired at the expense of stemness and thus reproductive capacity. In this scenario both the TSC and its progeny would die out if they were not to act as a cooperative unit. Viewed from an inclusive fitness perspective, the tumor as a whole enhances its reproductive fitness by cooperation and division of labor - that is the TSC subordinates its non-reproductive descendants by epigenetic programs that commit them to functional differentiation for altruistic behavior. Like social insect workers, non-reproductive cells increase their own fitness indirectly by "helping" the TSC to spread copies of their genes identical by descent via metastazation. Therefore, an important aim of research on tumor heterogeneity may be to decipher the algorithms that direct tumor self-construction by division of labor as allegorized by functional compartmentalization of superorganisms.

Colony members to some extent can switch tasks according to the context in a self-organizing manner [6,19]. This results in a highly adaptive functional ontogeny of temporal division of labor and task allocation that is maintained by haptic, pheromonal and chemotactic signals [5,6,63], and which is similarly present in neoplasms (e.g. as a complex bouquet of auto- and paracrine feedback loops) [22]. For instance, some tumor cells within breast cancer are known to stimulate their clonemates via secreted factors such as lysophosphatidic acid (LPA) [64] and epidermal growth factor (EGF) [65]. The response upon these factors in turn depends on the expression profile of cognate receptor(s) on the surface of the receiving tumor cell(s). Hence, albeit these ligands might be ubiquitously present throughout the entire tumor mass, only certain subsets of tumor cells might react on them as a functional compartment because they are epigenetically or genetically programmed to express the cognate receptor(s).

Moreover, the propensity of taking over certain tasks may correlate with the age of an individual. As colony members grow older, they proceed through a loosely defined series of labor roles (age-polyethism): those entail nursing of the queen and brood at first (close vicinity of young individuals to the reproductive), then housekeeping labor (nutrition, detoxification) elsewhere in the colony, and finally foraging outside [9,15,19]. Though genetic influences on caste differentiation [66] and division of labor have been documented [67-70], caste differences are usually based on epigenetic differences. Thus, as first suggested by Darwin, genes do not determine castes but caste plasticity responding to environmental conditions [71].

**Figure 2:**
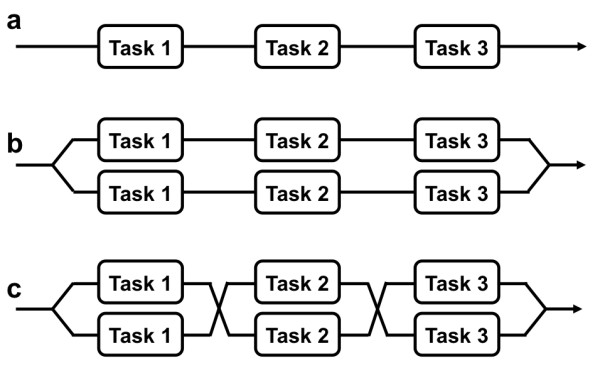
**Task switching and functional plasticity (adapted from **[6]**): **Part of the Darwinian success of superorganisms is the ability of the workers to switch tasks quickly and reliably. This can be understood as an issue of labor optimization: **A) **A non-social organism has no choice when addressing a task but to perform it as an *unbroken series of steps*. **B) **A colony can perform many such tasks simultaneously in *parallel series*. **C) **The whole process accelerates if the workers switch opportunistically from task to task to perform whatever task is closest in a *series-parallel process*, which is observed in some social insects. The efficacy of the system increases if groups of workers are specialized in size, anatomical proportions (allometry) and physiological competence (metabolic division of labor) to perform certain roles. This kind of task partitioning evidently decreases cost per unit yield in time and energy.

This suggests that genetic or epigenetic variations determine the sensitivity of an individual to specific proximate factors of the environment, which thereby guide the commitment to one or another caste [72], which is likely also true for tumors [73,74] (Figure 2).

Below we will highlight some specific analogies between tumors and superorganisms focusing mainly on how TSCs and their progeny benefit from division of labor:

## Angiogenesis

Social insect species with populous societies have evolved sophisticated strategies of shelter and alimentation. Workers construct tunnels and trail systems that guarantee constant oxygen and food supply to the colony's breeding core [5,6,63]. This is akin to specialized tumor cells that attract complex vascular networks and simultaneously induce sheltering fibrosis - a process termed heterotypic tumor/stroma interaction [29]. In addition, growing evidence suggests that within some cancers neoplastic cells differentiate into vessel-like "parenchyma". Angiogenic mimicry complements tumor-induced angiogenesis as a form of tumor metaplasia [29,75], a process that also applies to other forms of trans-differentiation (e.g. hormone production), which may present clinically as a paraneoplastic syndrome [76].

Although the degree to which cancer cells resemble endothelial cells is debatable, there is agreement that cancer cells can directly line the lumen of functional tumor blood vessels [77]. These cells, like the foragers in ant colonies, do not reproduce, but instead enable tumor growth indirectly by attraction of heterotypic tissues through chemotactic substances (e.g. VEGF) [29], as ants attract and recruit nestmates and even prey by odor trails and pheromones [5,6].

## Moving out

Sporadically, TSCs interrupt their notorious asymmetric cell cycling and produce other TSCs through symmetric cell division [78]. These new TSCs may differ from their differentiated clonemates not only in pluripotency, but also in possibly acquired traits for metastasis [79]. In ant colonies, metastasis is mirrored by young queens traveling to distant places within the ecosystem in search for a place suitable for establishing new breeding chambers [5,6]. Likewise, novel pluripotent TSCs disperse to new microenvironments within the body that harbor a "natural" proper niche (soil) [80].

Under natural conditions, solitary colony founding is by far the most dangerous phase in the life history of an individual queen, and a large percentage of young queens fall victim to predators or parasites. Social insects therefore usually delay the production of sexuals until they have reached a critical worker number at which the efficient production of large numbers of sexual offspring has become feasible [5,8]. In analogy, metastasis is often observed for the first time at late tumor stages, which have already reached a considerable size [81]. Our functional compartment model of a solid tumor as a superorganism suggests that very small tumors simply cannot afford the loss of cells through precocious metastasis, since they could not support the assembly of the early tumor niche, which would be very disadvantageous for the survival of the young primary colony.

Although metastasis will be lethal for most of the tumor cells, a very few will succeed in founding new colonies enabled by either acquired beneficial traits on their journey or pre-existing favorable factors of their own and/or the microenvironment [80]. Upon arrival, TSCs will start to reactivate intrinsic programs of asymmetric cell division to found a new colony that is a metastasis [82], while losing migratory activity like ant queens cast off their wings. In epithelial tumors this "spread and seed" is performed by the embryonic trait of epithelial-mesenchymal-transition and its reversal in mesenchymal-epithelial-transition [79]. During metastasis most metastasizing cells encounter new and possibly hostile environments (e.g. surrounding tissue, blood or lymphatic fluid), which may select for certain traits of the cells that allow survival in and colonization of other organs. Moreover, cells within already established metastases continue to underlie spontaneous (epi-)genetic mutations. Hence, metastasized cells often differ markedly from their parental primary tumor [83].

Interestingly, cancer cells may cooperate to change the microenvironment and ultimately found a new colony [84]. In analogy, in honeybees and many ant species new colonies are founded cooperatively by queens and workers by budding or fragmentation of the maternal colony [5,6,15]. Likely, TSCs also sporadically metastasize jointly with other non-reproductive cells (workers) in a coordinated fashion [84]. These TSC guardians may help to establish an early TSC niche at the distant and possibly hostile destination. Of note, this collective behavior of invading and metastasizing cancer cell populations has been recently also allegorized to swarm-like behavior of social insects [57], which may be the result of very similar coordinated processes of decision-making. In both systems only a very small proportion of actively invasive individuals - that is the proportion of "decision-makers" - is needed to cause a transition to collective and cohesive motion of a large body of followers [57,85]. Hence, identifying and targeting the functional compartment of decision-makers inducing metastasis in cancer may have profound clinical implications.

## Surveillance and immunoediting

Superorganisms developed sophisticated mechanisms to adapt and modify their environment and to cope with rivals. Several ant species feign death or camouflage themselves to confuse and repel predators. Others violently defend their territories in lethal battles, engage in elaborated attack maneuvers and/or build specialized nest constructions hampering intruders [5,6,15]. In analogy, also a malignant tumor has to evade from control mechanisms of the hosting organism in order to convey its parasitic growth. Consistently, there is broad evidence that tumors hijack features of immune cells, which were intended to attack the tumor, for their own purposes. For instance, some cancer cells specialize in recruiting immune cells like macrophages by secretion of platelet derived growth factor (PDGF), which in turn stimulates angiogenesis, fibrosis and ultimately metastasis by secretion of transforming growth factor beta (TGF-beta), EGF and receptor activator of NF-kappa-B ligand (RANKL) (for review see [22] and references therein).

These immuno-evasive features are thought to evolve during tumor evolution through the interplay of tumor cells and the innate and adaptive immunity:

Paul Ehrlich first proposed that transformed cells arise continuously within our bodies and that the immune system eradicates them before they may form a clinically apparent tumor [86]. Subsequently, experimental evidence by tumor transplantation models hinted to the existence of tumor-associated antigens and promoted the concept of immune surveillance [87]. Now it is accepted that tumor infiltrating lymphocytes (TILs) can attack and eradicate tumor cells. Accordingly, tumors must evolve mechanisms to escape immune control in a process called immunoediting, which consists of three phases [28]:

(1) *Elimination: *solid tumor of more than 2-3 mm require robust blood supply and stromal remodeling, which in turn induces subtle inflammation. The transformed cells can be recognized by recruited TILs that initiate a specific immune response [28].

(2) *Equilibrium: *The continuous sculpting of tumor cells selects immuno-resistant variants due to reduced immunogenicity (immune selection), which explains the apparent paradox of clinical tumor-formation in otherwise immunocompetent individuals [28].

(3) *Escape: *diverse tumor-derived factors including endothelial differentiation-related factor 1 (EDF1), VEGF, interleukin 10 (IL-10) and TGF-beta induce complex local and regional immunosuppressive networks. Although deposited at the primary site, these soluble factors extend immunosuppressive effects into local lymph nodes and the spleen, thereby facilitating invasion and metastasis [88,89]. According to our model of division of labor within a solid tumor, it is likely that those factors may only be secreted by specialized non-TSCs.

Although many cancers express specific antigens, immune surveillance appears inefficient. As some social insects reduce their visibility by elaborate camouflage techniques [6], tumor cells may elude immune control by downregulation of their major histocompatibility complexes (MHC) [90,91]. Likewise, the tumor stroma also has immuno-protective functions [92]: the tumor stroma (non-tumor cells and extracellular matrix) binds and obscures tumor antigens and thus competes with antigen-presenting cells for the antigen and additionally increases the intratumoral interstitial fluid pressure preventing immigration of immune effectors [93]. Hence, tumor cells that specialize in inducing fibrosis may contribute to the overall fitness of the tumor.

## Targeting algorithms of division of labor that direct self-construction of solid tumors

Though the details of division of labor and caste differentiation in insect societies are not completely understood, the processes involved have been described as social algorithms, i.e., epigenetic programs that can be regarded as an operating manual by which the colony assembles itself. Each step of the program is determined by decision rules that allow an individual to proceed on a defined pathway from one to the next decision point until the end of the sequence is reached. A complete sequence of such binary decisions is called an algorithm [6]. In analogy to social insects, an inherent epigenetic program may guide a tumor cell along a sequence of gradual differentiation towards a specific function that is relevant for the whole tumor or it may cause changes in the cell's behavior within its functional repertoire. Conditioned by the ongoing and simultaneous decisions of all cells, the tumor as a whole creates emergent patterns of adaptive responses to environmental conditions such as therapy and hypoxia [22,24,94]. The epigenetic program in each cell thereby defines how and upon which stimuli it will react.

According to our functional compartment model of solid tumors as superorganisms we can identify at least two major decision points of a TSC and its non-TSC derivatives: first the TSC has to decide, whether it will divide symmetrically and thus duplicate or divide asymmetrically and hence give rise to a more differentiated non-TSC that may help to establish a TSC niche.

**Figure 3:**
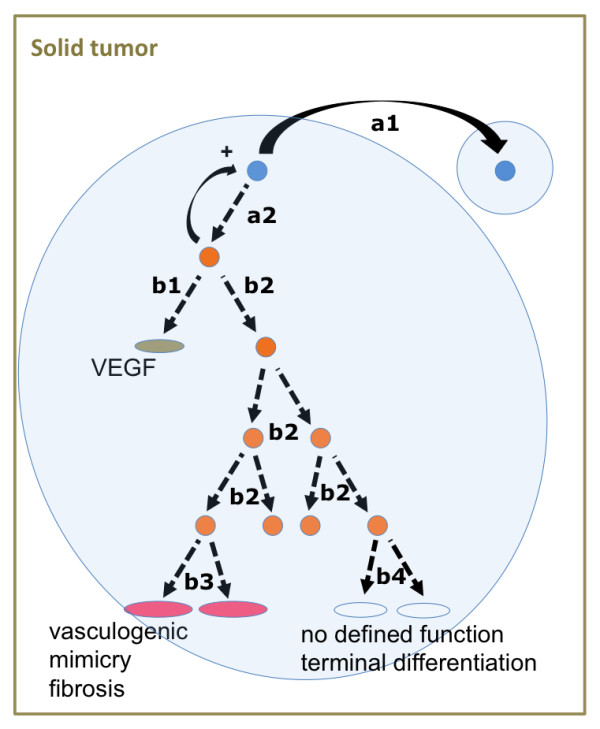
**Putative algorithm for tumor self-assembly and possible clinical interventions according to the functional compartment model**: Depicted is a schematic illustration of two colonies (blue circles) within a solid tumor (green box). At each cell division a TSC (blue) has to decide whether it will divide symmetrically (a1) or asymmetrically (a2). The resulting non-TSC from decision a2 has in turn the options to differentiate early (b1) and may thus gain functions like the production of growth factors and cytokines (e.g. VEGF) that potentially support the colony or to divide as a transitory amplifying cell several times (b2). In the latter scenario the non-TSC will differentiate and gain growth-supporting functions at a later time point (b3+b4). This theoretical model implies possible anti-cancer interventions: drugs that would specifically inhibit the TSC decision at point a1 or a2, such as "epigenetic therapeutics" [105], would obviously prevent outgrowth of a tumor. Conventional chemotherapy mostly affects fast proliferating cells (b2), but hardly targets slow-proliferating TSC and differentiated non-TSC [25]. Another option would be drugs that specifically inhibit the early differentiation (b1) or the function of already differentiated non-TSC (e.g. epigenetic [105] and/or antiangiogenic therapeutics [29,105,106]). Another approach is to drive non-TSC to terminal differentiation without any oncogenic function (b4), which is currently employed as a "differentiation therapy" in various cancers such as neuroblastoma and acute myeloid leukemia [107-109].

Within the second major step, a non-TSC has to decide whether it will divide as a transitory amplifying cell for the expense of delayed differentiation or whether it differentiates early to gain special functions such as attraction of blood vessels or induction of fibrosis. Of note, functional differentiation is not necessarily associated with morphological changes. Hence, tumor heterogeneity may be achieved by either functional and/or phenotypical differentiation [25] (Figure 3).

In analogy to a member of a certain caste within a superorganism, these algorithmic cellular fate decisions may be promoted by the cell's inherent sensitivity to specific inductive factors of the environment, e.g. the sensitivity to hypoxia, cytokines or other tumor cells. This sensitivity, which is private to the non-TSC, might be the result of an epigenetic program inherited from the cell's ancestral TSC at the time of asymmetric cell division.

## Technical approaches and perspectives

The analysis of plasticity of functional labor roles as epigenetic (and in the case of solid tumors also genetic) adaptations remains one of the outstanding challenges of socio- as well as tumor biology. But how might patterns of plasticity be conceptualized to advance the understanding of division of labor?

The advances in technology through the 1980s and 1990s allowed for more efficient separation of cells based on cell marker phenotypes, leading to the identification of normal hematopoietic stem cells in 1988 [95]. However, since then the major obstacles to identify, purify and to distinguish TSC from their differentiated derivatives mostly arise from the lack of robust markers [25]. Using resources such as array comparative genomic hybridization, expression sequence tags and microarrays [96-98], researchers may possibly identify novel factors that induce functional compartmentalization of individual tumor cells. The genomics era will succeed to scrutinize genetically complex patterns of functional traits controlled by multiple genes [99].

If we identified caste specific and thus functionally related cell surface markers, we would be able to sort and expand those cells *in vitro *and subject them to various functional assays such as drug-resistance screenings [25]. Moreover, those markers could be used to label distinct functional compartments in tumor tissue sections to enable microarray-based analysis of gene expression signatures in microdissected cells [100] of clinical specimens of the patients' tumors. These data could be further analyzed *in silico *to characterize gene expression patterns associated with drug response and prognosis. Functional characterization of those expression patterns would possibly distill *bona fide *targets for pharmaceutical high-through-put screenings such as the surface-plasmon-resonance technique for small molecule inhibitors, which has already lead to the identification of promising anti-cancer agents [101].

## Conclusions: lessons learned from superorganisms

Clinically, traits of functional compartmentalization and stemness correlate with metastatic disease and thus poor prognosis [102,103]. For decades, classical chemotherapy was directed against the highly proliferating progeny of TSCs. Slowly proliferating TSCs are, however, rarely affected and are nowadays accepted as the major cause of relapse [39,104].

Thinking of solid neoplasms as superorganisms with complex compartments and functions clarifies that a chemotherapeutic strategy addressing only the proliferating caste is not likely to succeed in eradicating all tumor cells in all compartments, as well as those on the move (G_0 _phase during metastasis). To kill an ant colony effectively it is not enough to simply kill the workers but the reproductive queen needs to be destroyed. Modern control products are designed to exactly do this [6]. Likewise, cancer therapies are most likely best targeted at the level of TSCs.

We think that beyond the targeted therapy of TSCs, though, modern anti-cancer therapies also need to include drugs specifically directed against non-TSCs that have functional relevance for the whole tumor (e.g. cells that promote angiogenesis/vasculogenic mimicry, fibrosis and immune escape). Thus, one important goal of research on tumor-heterogeneity is to understand the underlying algorithms and mechanisms of tumor sub-specialization. This will enable the development of novel concepts of targeted therapy, which will specifically attack each cohort of sub-specialized tumor cells (Figure 3).

Only if we succeed in identifying the underlying algorithms of the superorganism "solid tumor", we can elaborate complex, multilayered, and personalized therapy strategies, which can overcome the heterogeneous functional compartments and thus the tumor itself.

## Conflict of interest

The authors declare that they have no conflict of interest.

## Authors' contributions

TG and JH drafted and wrote the paper. TG designed the figures and the table. SH provided genetic, JH sociobiological, and TG and SB oncologic guidance. All authors read and approved the final manuscript.
